# Developmental Changes in *Peripherin-*eGFP Expression in Spiral Ganglion Neurons

**DOI:** 10.3389/fncel.2021.678113

**Published:** 2021-06-15

**Authors:** Karen L. Elliott, Jennifer Kersigo, Jeong Han Lee, Israt Jahan, Gabriela Pavlinkova, Bernd Fritzsch, Ebenezer N. Yamoah

**Affiliations:** ^1^Department of Biology, CLAS, The University of Iowa, Iowa City, IA, United States; ^2^Department of Otolaryngology, CLAS, The University of Iowa, Iowa City, IA, United States; ^3^Department of Physiology, School of Medicine, University of Nevada, Reno, Reno, NV, United States; ^4^Institute of Biotechnology, Czech Academy of Sciences, Vestec, Czechia

**Keywords:** peripherin, *Prph*-eGFP, type II spiral ganglion neurons, outer hair cells, cochlear nucleus

## Abstract

The two types of spiral ganglion neurons (SGNs), types I and II, innervate inner hair cells and outer hair cells, respectively, within the mammalian cochlea and send another process back to cochlear nuclei in the hindbrain. Studying these two neuronal types has been made easier with the identification of unique molecular markers. One of these markers, peripherin, was shown using antibodies to be present in all SGNs initially but becomes specific to type II SGNs during maturation. We used mice with fluorescently labeled peripherin (*Prph-*eGFP) to examine peripherin expression in SGNs during development and in aged mice. Using these mice, we confirm the initial expression of *Prph-*eGFP in both types I and II neurons and eventual restriction to only type II perikarya shortly after birth. However, while *Prph-*eGFP is uniquely expressed within type II cell bodies by P8, both types I and II peripheral and central processes continue to express *Prph-*eGFP for some time before becoming downregulated. Only at P30 was there selective type II *Prph-*eGFP expression in central but not peripheral processes. By 9 months, only the type II cell bodies and more distal central processes retain *Prph-*eGFP expression. Our results show that *Prph-*eGFP is a reliable marker for type II SGN cell bodies beyond P8; however, it is not generally a suitable marker for type II processes, except for central processes beyond P30. How the changes in *Prph-*eGFP expression relate to subsequent protein expression remains to be explored.

## Introduction

The mammalian auditory sensory organ, the cochlea, contains the neurosensory cells specific for the transduction and transmission of sound stimuli to the brain. Bipolar spiral ganglion neurons (SGNs) innervate two different types of mechanosensory hair cells at the periphery and send a process centrally to cochlear nuclei in the hindbrain (De No, 1981). The two types of hair cells, inner hair cells (IHCs) and outer hair cells (OHCs), are innervated by two types of SGNs, myelinated type I and unmyelinated type II, respectively (Brown et al., 1988; Hafidi, 1998; Nayagam et al., 2011). While type I SGNs are the primary auditory neurons for encoding sound stimuli, the function of type II SGNs is to summate and integrate OHC activity to confer cochlear sensitivity and tuning (Guinan and Gifford, 1988). Even though OHCs outnumber IHCs threefold, their innervation by type II SGNs constitutes only about 5–8% of total auditory afferent neurons; the remaining approximately 95% of neurons are type I that innervate IHCs (Spoendlin, 1971). The pattern of innervation delineates the inequality in numbers between the two types of SGNs. A single IHC is innervated by multiple type I fibers, whereas a single type II neuron may innervate 10 or more OHCs (Hafidi, 1998; Simmons and Liberman, 1988; Rubel and Fritzsch, 2002; Weisz et al., 2009; Coate and Kelley, 2013). Centrally, both types I and II SGNs project to three regions of the cochlear nucleus: the anteroventral cochlear nucleus (AVCN), posteroventral cochlear nucleus (PVCN), and the dorsal cochlear nucleus (DCN), maintaining the tonotopic organization reflected in the cochlea (Brown et al., 1988; Nayagam et al., 2011; Fritzsch et al., 2019).

Studying the two different types of SGNs and their projections has been made easier by discovering unique molecular markers (Petitpre et al., 2018; Shrestha et al., 2018; Sun et al., 2018). The prevailing type II neuronal markers are tyrosine hydroxylase (*Th*), calcitonin-related polypeptide alpha (*Cgrp*α), and nerve growth factor receptor (*Ngfr*). However, these markers are not exclusive to type II SGNs, as they have been shown to overlap with type I SGN expression (Vyas et al., 2017, 2019; Wu et al., 2018). In addition to these markers, an intermediate filament protein, peripherin, is exclusively expressed in mature type II SGNs (Hafidi, 1998; McLenachan et al., 2008; Froud et al., 2015). Peripherin has also been identified in select groups of sensory, motor, and autonomic neurons (Escurat et al., 1990); however, its exact function remains controversial (Lariviere and Julien, 2004). Most of the work on peripherin in type II SGNs has been studied using antibodies. Initially, peripherin protein was shown to be present in all SGNs but becomes restricted to type II SGNs during maturation after birth (Hafidi et al., 1993). Peripherin labeling was detected in both the soma and peripheral and central processes of type II SGNs (Hafidi et al., 1993; Hafidi, 1998; Maison et al., 2016). While antibody work has uncovered some information regarding peripherin protein expression in type II SGNs; not much is known about how *peripherin* expression changes over time.

To consolidate past research and to expand the range of studies from embryos to adults, we aim to provide a detailed investigation of *peripherin* expression in type II SGNs during development and aging by demonstrating their peripheral and central projections over time. For this, we used mice with fluorescent labeling of peripherin (*Prph-*eGFP). Our data show a delayed selective identification of type II neurons, as expected. Furthermore, approximately 2 weeks after birth, while the type II cell bodies retain intense labeling, the peripheral processes begin progressively losing *Prph-*eGFP expression. In the brain, we show an initial moderate *Prph-*eGFP expression in the central projection of SGNs that reduces in expression with age.

## Materials and Methods

### Mice

To examine *peripherin* expression over time in the murine auditory system, *peripherin*-EGFP (*hPRPH1-G*) genomic reporter transgenic mice were used (McLenachan et al., 2008). This *peripherin*-eGFP (*Prph*-eGFP) transgenic mice were backcrossed to the CBA/CaJ background (Erway et al., 1996; Johnson et al., 2000). PCR confirmed genotyping on tail DNA with the following primers: B10Screen5b 5′-TGCCAGGACCCCACCATTTC-3′, B10Screen3b 5′-AGCTGAGACTACAGGCGCGTGCCA-3′, and EGFP-ProbeR 5′-GACAACCACTACCTGAGCACCCAGT-3′.

To visualize both types I and II peripheral processes in the cochlea at E18.5, we used the Neurod1-cre; tdTomato transgenic construct (Neurod1-cre, Jackson Laboratory stock #028364; tdTomato, Jackson Laboratory stock #007914). PCR confirmed genotyping on tail DNA with the following primers: tdTomato—IMR9105 5′-CTGTTCCTGTACGGCATGG-3′, IMR 9103 5′-GGCATTAAAGCAGCGTATCC-3′, IMR9020 5′-AAGG GAGCTGCAGTGGAGTA-3′, and IMR9021 5′-CCGAAAATC TGTGGGAAGTC-3′; and Neurod1-cre—IMR0042 5′-CTAGG CCACAGAATTGAAAGATCT-3′, IMR0043 5′-GTAGGTGGA AATTCTAGCATCATCC-3′, CRE1 5′-CCTGTTTTGCACGTT CACCG-3′, and CRE3 5′-ATGCTTCTGTCCGTTTGCCG-3′.

All animal work was performed as required by the United States Animal Welfare Act and the National Institutes of Health’s policy to ensure proper care and use of laboratory animals for research and under established guidelines, supervision, and approved protocols by the Institutional Animal Care and Use Committee (IACUC) of The University of Nevada, Reno, and The University of Iowa.

### Fixation and Tissue Preparation

Mice were anesthetized, culled, and transcardially perfused at various stages (E18.5, P4, P7, P8, P15, P30, P40, and 9 Mo) with 4% paraformaldehyde in phosphate-buffered saline (PBS) (pH 7.6) with 0.3 M sucrose to maintain neuronal structural integrity (Fritzsch, 1979; Cragg, 1980). The head was removed and shipped in 0.4% paraformaldehyde (PFA) with 0.3 M sucrose on ice protected from light. The head was then bisected, and the brain halves and temporal bones were removed. The brain was cryoprotected in 30% sucrose overnight, embedded in Tissue-Tek OCT medium (Sakura Finetek Inc., 4583), and quick frozen in a dry ice ethanol bath. The sample blocks were wrapped in foil and stored briefly at –80°C until sectioning. To section, the blocks were acclimated to –20°C, trimmed, and mounted on the specimen holder of a Leica CM1800 cryostat with OCT. The sample was sectioned coronally at a thickness of 50–60 μm, and the sections were collected on Superfrost Plus slides (Thermo Fisher Scientific 12-550-15). The slides were stored briefly at –80°C until ready to view. To view, the slides were washed in PBS for approximately 4 min to remove the embedding medium and coverslipped using Fluoromount-G with DAPI (Thermo Fisher Scientific 00-4959-52). Care was taken to protect the samples from light at all procedural stages.

The temporal bones were decalcified in 0.25 M ethylenediaminetetraacetic acid (EDTA) solution (RPI E57020) for up to 5 days (>P8) with daily solution changes. Decalcified cochleae were washed in PBS and microdissected, and the tectorial membrane was removed. Cochlear turns were flat mounted in glycerol or Fluoromount-G with 4′,6-diamidino-2-phenylindole (DAPI) for viewing.

### Immunofluorescence

Whole-mount, dissected cochleae were blocked and permeabilized with 5% NGS (Sigma-Aldrich G9023) in PBST (PBS + 0.5% Triton X-100) for 1 h then incubated in primary antibody solution (PBS + 0.1% Triton X-100 + antibodies) for 24–48 h at 4°C. Primary antibodies used were as follows: rabbit anti-Myosin-VIIa (Myo7a; Proteus BioSciences 25-6790, 1:300), mouse antineuronal nuclear antigen (NeuN; Millipore MAB377, 1:500), and rabbit antiperipherin (Millipore AB1530, 1:100). After several PBS washes (3 × 1 h) at room temperature, the samples were incubated in species-specific secondary antibody solution at 4°C for 12–24 h. The secondary antibodies used were as follows: Alexa Fluor 488 goat antirabbit immunoglobulin G (IgG) (Thermo Fisher Scientific A11008, 1:500), Alexa Fluor 647 goat antirabbit IgG (Thermo Fisher Scientific A32733, 1:500), and Alexa Fluor 647 goat antimouse IgG (Thermo Fisher Scientific A11007, 1:500). Hoechst 33258 (Thermo Fisher Scientific H1399, 1:2,000) or DAPI (Sigma-Aldrich D9542, 1 μg/ml) nuclear counterstain was used in some samples. Finally, the samples were washed several times (3 × 1 h) in PBS before viewing.

### Imaging

Images were acquired using a Leica SP8 scanning laser confocal microscope, analyzed with Leica LAS X software and processed with CorelDRAW graphics suite. Images were taken at 1–6-μm thick optical sections to compile a given stack, in up to four different colors (405, 488, 552, and 638 nm laser lines) using three different magnifications (10× with a 0.6 NA; 20× with a 0.95; 63× with a 1.4 NA).

### Quantification

For analysis of the distribution of peripherin-positive neurons within the cochlea, we quantified the number of peripherin-positive neurons in the proximal, middle, and distal regions for the apex, middle, and base. Three 150-μm wide boxes were drawn randomly around the spiral ganglia region, each for the base, middle turn, and apex per animal. The box’s length was the distance from the most proximal SGN to the most distal SGN cell body. Each box was divided into equal thirds, separating the proximal, middle, and distal regions, and the number of *Prph-*eGFP-positive neurons was manually counted within each region. We quantified four P15, four P30, and three 9-months-old cochleae.

For the analysis of peripherin labeling within the hindbrain, the peripherin signaling intensity level was quantified within the AVCN, PVCN, DCN, and the vestibular nucleus, adjacent to its entry point E18,5, P4, P30, and 9-months-old mice. Three 100 × 100 μm boxes were drawn at random within maximum projection images of coronal sections of the AVCN, PVCN, DCN, vestibular nucleus, and trigeminal nucleus. This size was selected as a compromise between maximizing the area quantified and having the box fit within the different brain regions’ boundaries. The trigeminal nucleus was chosen. It had a high level of peripherin expression throughout the entire nucleus and was located within each coronal section containing the AVCN, PVCN, DCN, or vestibular nucleus. In addition, three 100 × 100 μm boxes were drawn randomly in areas of background per image. Images were cropped along the boxes in CorelDraw and exported as individual TIFF files. Individual TIFF files were analyzed using the histogram function in ImageJ software. The resulting analysis calculates the mean fluorescent intensity of the entire TIFF file. The background’s mean fluorescent intensity was subtracted from respective images of the AVCN, PVCN, DCN, vestibular nucleus, and trigeminal nucleus. The fluorescent intensity of the trigeminal nucleus (minus background) was set at 100%, and the fluorescent intensities of the AVCN, PVCN, DCN, and vestibular nucleus (minus backgrounds) were calculated as a percent of the portion of the trigeminal nucleus from the image in which they were obtained. The mean intensities were averaged, and the standard error was calculated using Microsoft Excel. Statistical significance was performed with one-way ANOVA with the Tukey honestly significant difference (HSD) *post hoc* test. The confidence level was set at 95%.

## Results

### Spiral Ganglion Neurons

**FIGURE 1:**
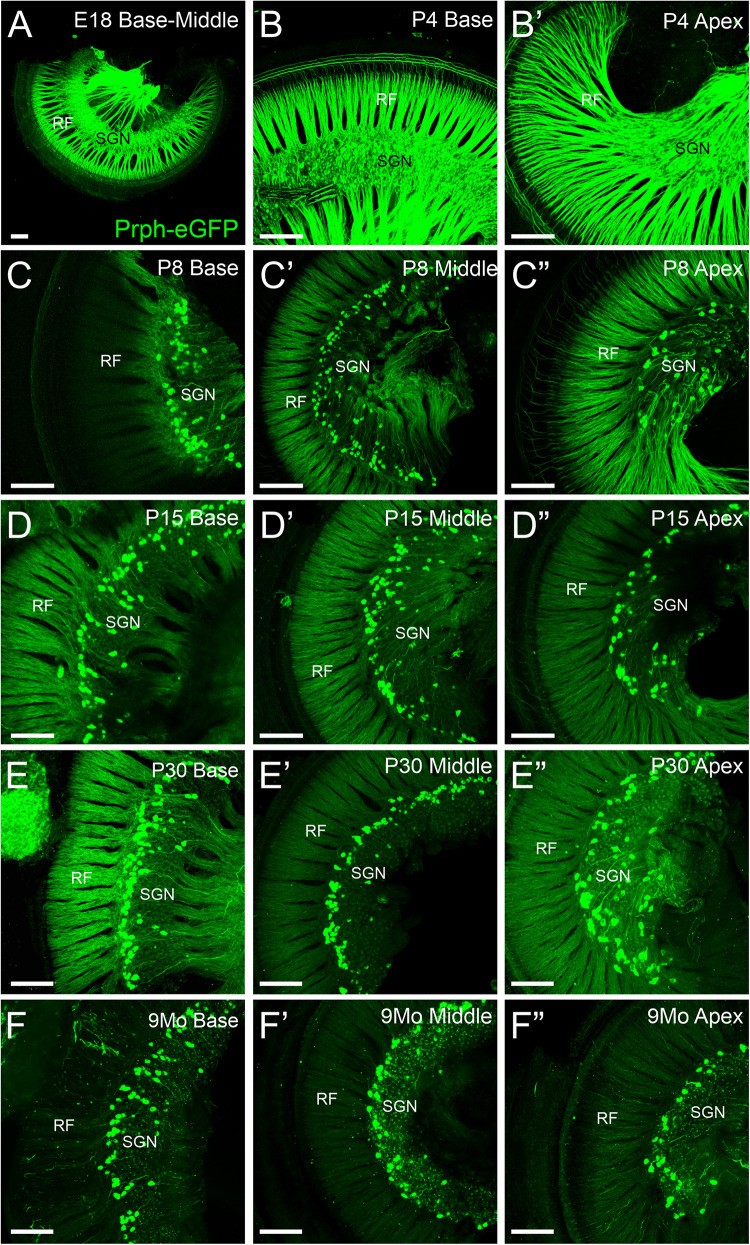
Expression of *Prph-*eGFP in spiral ganglion neuron (SGN) cell bodies in the spiral ganglia. **(A)** Cochlea from an E18.5 mouse showing *Prph-*eGFP expression (green) in all SGNs. **(B,B′)** Cochlea from a P4 mouse showing *Prph-*eGFP expression in most SGNs at the base in panel **(B)** and apex in panel **(B′)**. **(C–C″)** Cochlea from a P8 mouse showing *Prph-*eGFP expression restricted to only a subset of cells, the type II SGNs at the base in panel **(C)**, middle turn in panel **(C′)**, and apex in panel **(C″)**. **(D–D″)** Cochlea from a P15 mouse showing *Prph-*eGFP expression restricted to only a subset of cells, the type II SGNs at the base in panel **(D)**, middle turn in panel **(D′)**, and apex in panel **(D″)**. **(E–E″)** Cochlea from a P30 mouse showing *Prph-*eGFP expression restricted to only a subset of cells, the type II SGNs at the base in panel **(E)**, middle turn in panel **(E′)**, and apex in panel **(E″)**. **(F–F″)** Cochlea from a 9-months mouse showing *Prph-*eGFP expression restricted to only a subset of cells, the type II SGNs at the base in panel **(F)**, middle turn in panel **(F′)**, and apex in panel **(F″)**. Note the expression of *Prph-*eGFP-positive cells is primarily at the distal region of the spiral ganglia, adjacent to the radial fibers (RF) in P15, P30, and 9-months mice, especially at the base and middle turn. Scale bars are 100 μm.

All SGN cell bodies are initially positive for *Prph-*eGFP at E18.5 (Figure 1A), consistent with previous work using peripherin antibodies (Hafidi et al., 1993; Hafidi, 1998; Nayagam et al., 2011). The level of *Prph-*eGFP remains relatively high in all SGNs at P4 (Figures 1B,B′), although some SGNs begin to have less expression than others (Figure 3C). At P8, *Prph-*eGFP has become restricted to the type II SGNs, with no expression observed in type I cell bodies (Figures 1C–C″). These type II cell bodies continue to express *Prph-*eGFP through at least 9 months of age (Figures 1D–F″).

**FIGURE 2:**
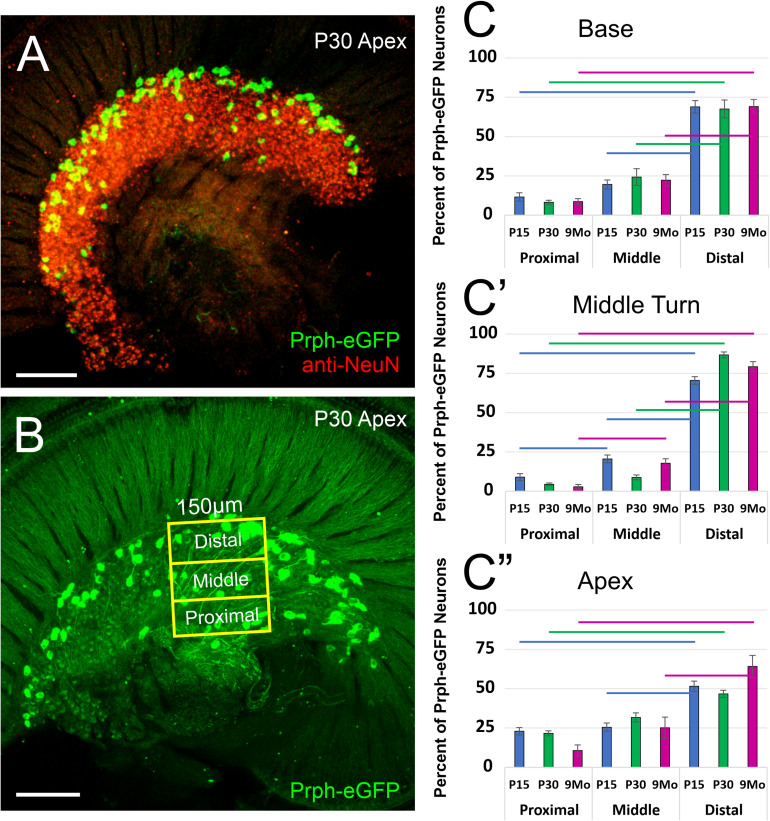
Quantification of the distribution of *Prph-*eGFP-positive spiral ganglion neurons (SGNs). **(A)** *Prph-*eGFP-positive cells (green) are located along the more distal end of the spiral ganglion, of which all SGN nuclei were labeled with an antibody against NeuN (red). **(B)** Schematic shows how the number of *Prph*-eGFP-positive cells within representative regions of the spiral ganglion, proximal, middle, and distal, was quantified. **(C–C″)** Means and standard errors of the means of the percent of *Prph-*eGFP-positive neurons present within each subregion: proximal, middle, or distal, as compared with the total number of *Prph*-eGFP-positive neurons within the whole boxed area, at the base in panel **(C)**, mid-turn in panel **(C′)**, and apex in panel **(C″)** at P15 (blue, *n* = 4), P30 (green, *n* = 4), and 9 months (magenta, *n* = 3). Colored horizontal bars represent significant differences (*p* < 0.01). Scale bars are 100 μm.

These *Prph-*eGFP-positive type II cell bodies are not uniformly located throughout the spiral ganglion. Instead, these *Prph-*eGFP-positive cell bodies appear to be more concentrated along the distal region of the spiral ganglion (Figures 1C–F″), consistent with reports using antibody labeling (Hafidi, 1998; Nayagam et al., 2011; Defourny et al., 2013; Grandi et al., 2020). This asymmetrical distribution was better visualized with NeuN labeling of *Prph-*eGFP mice to label all SGN nuclei (Figure 2A). To confirm that these *Prph-*eGFP-positive type II SGNs are indeed asymmetrically located, we quantified the number of *Prph-*eGFP-positive neurons within 150-μm-wide representative areas of the spiral ganglia, dividing each area into proximal, middle, and distal regions for P15, P30, and 9-month animals (Figure 2B). This was repeated for each area of the cochlea: base, middle turn, and apex. For the base, middle turn, and apex of all three ages, there were significantly more *Prph-*eGFP-positive cells in the distal third than in the proximal third regions (ANOVA, *p* < 0.01, *n* = 3–4 animals with three measurements per animal) (Figures 2C–C″). In addition, for the base and middle turn, there were significantly more *Prph-*eGFP-positive cells in the distal third as compared with the middle-third regions for all ages (ANOVA, *p* < 0.01, *n* = 3–4 animals with three measurements per animal) (Figures 2C,C′). However, the asymmetrical distribution of *Prph-*eGFP-positive cells was less defined at the apex as compared with the near absence of *Prph-*eGFP-positive cells in the proximal or middle regions at the base or middle turn (Figures 1C–F″, 2). While there was a significant difference between distal and proximal regions in all ages, only at P15 and 9 months was there a significant difference between the distal and middle regions in the apex (ANOVA, *p* < 0.01, *n* = 3–4 animals with three measurements per animal, Figure 2C″). Interestingly, unlike the asymmetrical distribution of *Prph-*eGFP-positive cells in the spiral ganglion, there is a wide distribution of *Prph-*eGFP-positive cells in the vestibular ganglion (Supplementary Figure 1), suggesting that there may be some significance to the distribution.

**FIGURE 3:**
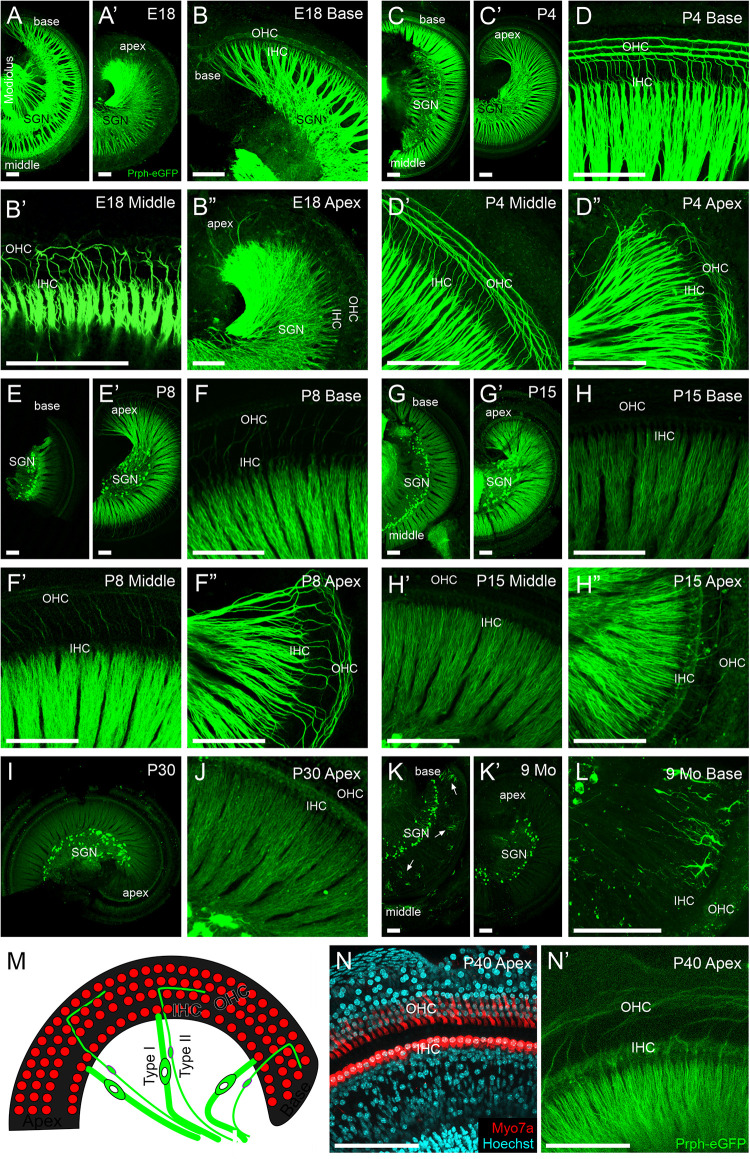
Peripheral projections of *Prph-*eGFP-positive neurons. **(A–B″)** E18.5 cochlea showing a high level of *Prph-*eGFP expression in peripheral processes to inner hair cells (IHC) and most outer hair cells (OHC). *Prph-*eGFP-positive processes to IHCs can be observed at the base in panel **(B)**, middle turn in panel **(B′)**, and apex in panel **(B″)**, whereas *Prph-*eGFP-positive processes to OHCs are only observed in the base in panel **(B)** and mid-turn in panel **(B′)**, not yet in the apex in panel **(B″)**. **(C–D″)** P4 cochlea showing a high level of *Prph-*eGFP expression in all peripheral processes. At this stage, type I and II peripheral processes to IHCs and OHCs, respectively, express robust levels of *Prph-*eGFP throughout the base in panel **(D)**, middle turn in panel **(D′)**, and apex in panel **(D″)**. **(E–F″)** P8 cochlea showing a high level of *Prph-*eGFP expression in processes to all IHCs and apical OHCs. At this stage, type I processes to IHCs express a strong level of *Prph-*eGFP throughout the cochlea in panel **(F–F″)**; however, type II processes to the basal in panel **(F)** and middle turn in panel **(F′)** OHCs express less *Prph-*eGFP than processes to apical OHCs in panel **(F″)**. **(G–H″)** P15 cochlea showing a decrease in *Prph-*eGFP expression in all processes at the base and middle turn. At this stage, both type I and II processes to IHCs and OHCs, respectively, in the base in panel **(H)** and mid-turn in panel **(H′)** express lower levels of *Prph-*eGFP. In the apex, the Prph-eGFP expression level remains high in type I processes to IHCs but has decreased in type II processes to OHCs in panel **(H″)**. **(I,J)** P30 cochlea showing a reduced expression *Prph-*eGFP. At this stage, both type I and II peripheral processes to IHCs and OHCs, respectively, show low expression levels of *Prph-*eGFP at the apex in panel **(J)**. **(K–L)** Nine-months cochlea showing almost no expression of *Prph-*eGFP in peripheral processes. At this stage, only the cell bodies retain *Prph-*eGFP expression; however, in the base, ramified cells of unknown origin were observed in panel **(L)**. **(M)** Diagram of the organ of Corti showing the location of IHCs and OHCs (red) as well as their innervation from type I and II SGNs (green), respectively. **(N)** Myo7a antibody labeling (red) and Hoechst nuclear staining (cyan) to show the location of IHCs and OHCs within the cochlea of an approximately 1-month-old mouse. **(N′)** Same cochlea as N showing *Prph-eGFP*-positive SGNs projecting stereotypically to the regions of the IHCs and OHCs. Scale bars are 100 μm.

In summary, these results show a significant asymmetrical distal distribution of mature *Prph-*eGFP-positive type II SGNs in all cochlea regions, with a more robust distal distribution in the base and middle turn.

### Peripheral Processes to Hair Cells

We next examined the peripheral processes to the hair cells in *Prph-*eGFP-positive neurons (Figure 3). At E18.5, peripheral processes of both type I and II neurons strongly express *Prph-*eGFP (Figures 3A–B″). *Prph-*eGFP-positive processes are observed projecting to OHCs only in the base and middle turn at E18.5 (Figures 3B–B″). Using a tdTomato reporter line driven by *Neurod1-cre*, we labeled all type I and II SGNs and showed that while there is no *Prph-*eGFP labeling yet to OHCs in the apex (Figure 3B″), the type II SGNs innervate these OHCs at E18.5 (Supplementary Figure 2), suggesting a delay in *Prph-*eGFP expression after hair cell innervation. *Prph-*eGFP-positive processes to IHCs can be seen in each region of the cochlea at this stage (Figures 3B–B″). Four days after birth, the peripheral processes of both type I and II neurons have maintained strong expression of *Prph-*eGFP (Figures 3C–D″). In addition, at P4, *Prph-*eGFP-positive processes to OHCs are now observed at the apex in addition to the base and middle turn (Figures 3D–D″). *Prph-*eGFP-positive processes to IHCs are observed in all areas of the cochlea as well (Figures 3D–D″). We note that at P7, selective antiperipherin-positive peripheral processes can be observed (Supplementary Figure 3), consistent with previous work showing peripherin antibody expression in postnatal stages (Hafidi, 1998; Vyas et al., 2017). At P8, while type I processes to IHCs express a strong level of *Prph-*eGFP throughout the cochlea, type II processes to basal and middle turn OHCs express less *Prph-*eGFP than type II processes to apical OHCs (Figures 3E–F″). By P15, the relative level of *Prph-*eGFP expression in both type I and II peripheral processes at the base and middle turn is less than that in the type II cell bodies, with expression in type II to OHCs being less than in type I to IHCs. Furthermore, expression of *Prph-*eGFP in type II peripheral processes to apical OHCs has also decreased some, although the expression of *Prph-*eGFP in type I SGNs to IHCs remains high (Figures 3G–H″). The intensity of expression in type I apical peripheral processes appears similar to that in type II *Prph-*eGFP-positive cell bodies (Figure 3G′). By P30, however, the level of *Prph-*eGFP in type I SGNs to IHCs in the apex has decreased (Figures 3I,J). There is minimal *Prph-*eGFP expression in peripheral processes at 9 months, and labeled type II processes out to OHCs were not observed (Figures 3K,L). Interestingly, there are highly ramified cells of unknown origin located among the peripheral processes at the base in the 9-months cochlea that express high levels of *Prph-*eGFP (Figure 3L).

Together, these results suggest that *Prph-*eGFP expression in the peripheral processes progresses in a basal to apical wave, beginning first with its upregulation at late embryonic stages, followed by its downregulation approximately 2 weeks later, the onset of hearing. After this point, the level of *Prph-*eGFP in the peripheral processes continues to decrease until it is at a barely detectable level compared with that in the cell bodies by 9 months.

### Central Projections of Spiral Ganglion Neurons

**FIGURE 4:**
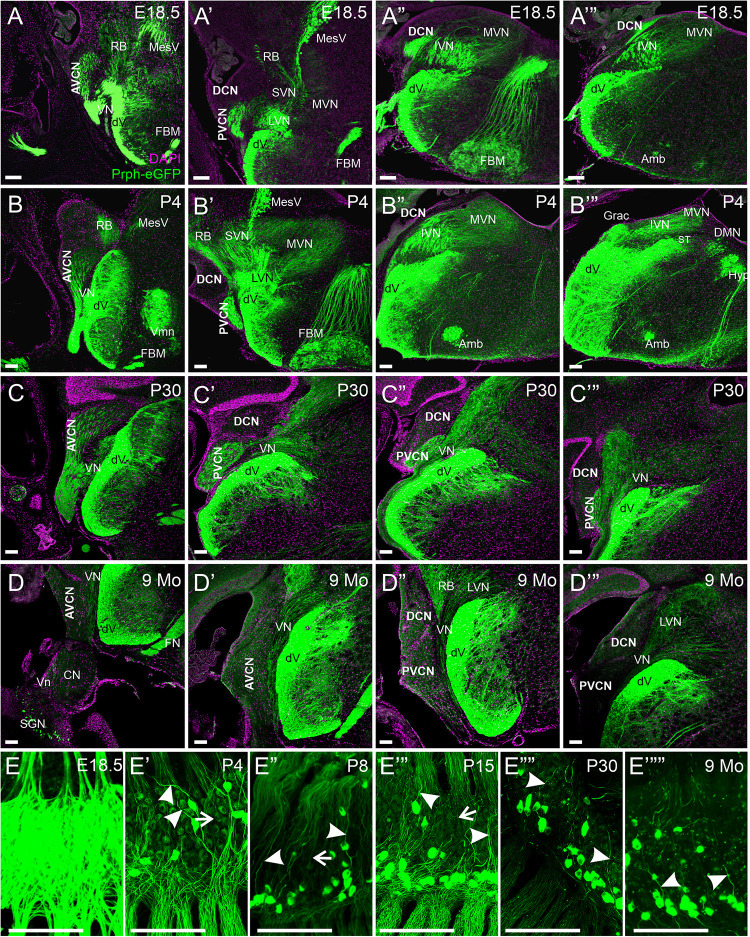
Expression of *Prph-*eGFP in SGN central projections. **(A–A″′)** Coronal sections of an E18.5 brain showing the projection of *Prph-*eGFP-positive neurons (green). At this stage, *Prph-*eGFP-positive neurons project to the AVCN, PVCN, and DCN. **(B–B″′)** Coronal sections of a P4 brain showing the projection of *Prph-*eGFP-positive neurons. At this stage, projections to the DCN express very little *Prph-*eGFP. **(C–C″′)** Coronal sections of a P30 brain showing the projection of *Prph-*eGFP-positive neurons. **(D–D″′)** Coronal sections of a 9-month brains showing the projection of *Prph-*eGFP-positive neurons. By 9 months, very little *Prph-*eGFP expression is found in projections to the AVCN, PVCN, or DCN, while projections to the vestibular nucleus retain some *Prph-*eGFP expression. **(E–E″″′)** Spiral ganglia of E18.5, P4, P8, P15, P30, and 9-months mice, respectively, showing the progression of *Prph-*eGFP expression from all central fibers (top of images) in E18.5, to selective expression in P30 and 9 months. Central processes of type II SGNs are labeled with arrowheads, and those of type I are marked with arrows. AVCN, anteroventral cochlear nucleus; PVCN, posteroventral cochlear nucleus; DCN, dorsal cochlear nucleus; Vn, vestibular nerve; VN, vestibular nucleus; MVN, medial vestibular nucleus; SVN, superior vestibular nucleus; IVM, Inferior vestibular nucleus; LVN, lateral vestibular nucleus; dV, descending tract of the trigeminal nucleus; MesV, the mesencephalic nucleus of the trigeminal; Vmn, trigeminal motoneurons; RB, the restiform body; ST, solitary tract; Grac, gracile; CN, cochlear nerve; SGNs, spiral ganglion neurons; DMN, dorsal motor neurons; Hyp, hypoglossal somatic motoneurons; Amb, ambiguous branchial motoneurons; FBM, facial branchial motoneurons. Magenta color is 4′,6-diamidino-2-phenylindole (DAPI) to show cell bodies. Scale bars are 100 μm.

Next, we examined the central processes of the cochlear nuclei in *Prph-*eGFP-positive SGNs. At E18.5, *Prph-*eGFP-positive processes were observed in each of the three regions of the cochlear nucleus, AVCN, PVCN, and the DCN (Figures 4A–A’″). However, by P4, the *Prph-*eGFP-positive projections to the DCN were noticeably downregulated compared with E18.5 and compared with the AVCN and PVCN, suggesting that *Prph-*eGFP expression is downregulated or the *Prph*-eGFP is no longer being transported along with central processes to the DCN (Figures 4B–B″). Similarly, at P30, while the AVCN and PVCN receive input from processes expressing *Prph-*eGFP, the DCN does not (Figures 4C–C″′). However, by 9 months, central processes to all regions of the cochlear nucleus do not express *Prph-*eGFP (Figures 4D–D″′). In contrast to SGNs, *Prph-*eGFP-positive vestibular neurons were observed projecting to vestibular nuclei and trigeminal neurons to trigeminal nuclei at all stages (Figure 4). The latter expressed very strong levels of *Prph-*eGFP throughout development and in the aged, 9-months mice. Given the *Prph-*eGFP labeling in both type I and II peripheral processes beyond when the *Prph-*eGFP labeling is confined to only type II cell bodies, we cannot be certain of the central processes’ origin. However, specific labeled *central* processes of these labeled type II cells can be observed by P30 and remains at 9 months (Figures 4E″″,E″″′), suggesting that after this time point, the *Prph-*eGFP-positive central processes are most likely type II fibers. Between P4 and P30, while type II SGNs have a stronger expression of *Prph-*eGFP, lower expression levels are observed in central processes of type I SGNs (Figures 4E′–E″′). At E18.5, all central processes are strongly labeled (Figure 4E).

**FIGURE 5:**
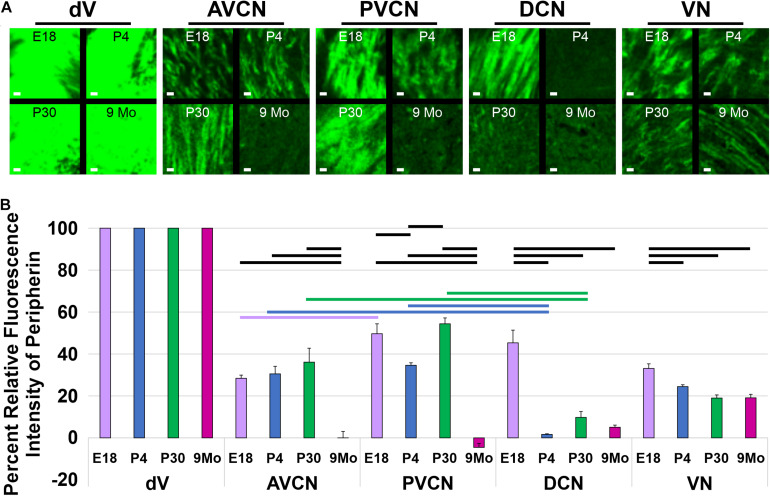
Quantification of *Prph-*eGFP positivity in different hindbrain regions. **(A)** Representative 100 × 100 μm areas within each of the brain regions from which the fluorescence intensity level of *Prph-*eGFP expression was quantified: trigeminal nucleus (dV), anteroventral cochlear nucleus (AVCN), posteroventral cochlear nucleus (PVCN), dorsal cochlear nucleus (DCN), and the vestibular nucleus (VN). Images were acquired from coronal sections of E18.5, P4, P30, and 9-month brains, as shown in Figure 4, and three 100 × 100 μm boxed areas per region were used for fluorescence intensity quantification. **(B)** Relative fluorescence intensities of *Prph-*eGFP in each brain region normalized to the trigeminal nucleus, set at 100% fluorescent intensity. Fluorescent intensities for the AVCN, PVCN, DCN, and VN are shown as percentages of the total fluorescence intensity in the trigeminal nucleus. Bold horizontal bars represent significant differences (*p* < 0.05). Scale bars are 10 μm.

To confirm these observational differences between the different regions of the cochlear nucleus and the vestibular nucleus across development and aging, we determined the mean fluorescent intensity for sample regions within the AVCN, PVCN, DCN, and vestibular nucleus adjacent to its entry point. Since neurons within the trigeminal nucleus expressed high levels of *Prph-*eGFP continuously, we used it as our reference point. We calculated the relative percent of fluorescent intensity of afferents innervating the AVCN, PVCN, DCN, and vestibular nucleus relative to the trigeminal nucleus for each time point (Figure 5). In the AVCN, the level of expression of *Prph-*eGFP remained relatively unchanged in early development, through at least P30, but by 9 months, expression was significantly lower (ANOVA, *p* < 0.05, *n* = 3 measurements; Figure 5B). Similarly, the expression of *Prph-*eGFP in the PVCN at 9 months was significantly lower than the other three earlier time points (ANOVA, *p* < 0.05, *n* = 3 measurements; Figure 5B). However, in the DCN, the level of *Prph-*eGFP significantly dropped at a much earlier time point of P4 (ANOVA, *p* < 0.05, *n* = 3 measurements; Figure 5B). While the level of *Prph-*eGFP in the vestibular nucleus also significantly dropped by P4 (ANOVA, *p* < 0.05, *n* = 3 measurements), this decrease was markedly less than in the DCN (Figure 5B). We next wanted to compare differences between the different cochlear nuclei within a given age. At E18.5, there was a slightly significant difference between *Prph-*eGFP expression between the AVCN and PVCN but no significant difference between either of those with the DCN (ANOVA, *p* < 0.05, *n* = 3 measurements; Figure 5B). At P4 and P30, the Prph-eGFP expression levels within the AVCNs and PVCNs were significantly higher than that in the respective DCNs (ANOVA, *p* < 0.05, *n* = 3 measurements; Figure 5B), although by 9 months, the level of *Prph-*eGFP was at an equally low level in all three areas of the CN (Figure 5B).

Together these results suggest that, like in the peripheral processes to the hair cells, central processes of SGNs to the cochlear nuclei downregulate the expression of *Prph-*eGFP by 9 months; however, the specific timeline of when this happens for projections to a given region of the cochlear nucleus is variable.

## Discussion

Our data expand upon previous studies using antibodies (Hafidi et al., 1993; Hafidi, 1998; Nayagam et al., 2011) to describe the gain and loss of *Peripherin* expression across several time points in development and 9-months-old mice. Using the *Prph-*eGFP mouse line (McLenachan et al., 2008), we examined expression in SGN cell bodies, their distal processes to hair cells, and the central projections to the cochlear nuclei. Our results here confirm previous antibody labeling, which demonstrated selective peripherin labeling of mature type II SGNs (Hafidi et al., 1993). In our study, *Prph-*eGFP becomes restricted to type II SGN cell bodies between P4 and P8 (Figure 1). Similarly, peripherin antibody staining also becomes restricted to type II SGN cell bodies after P3 in the rat (Hafidi et al., 1993). As in this previous study with peripherin antibodies, once *Prph-eGFP* became restricted, specifically type II SGN cell bodies, they remained restricted throughout adulthood (Figure 1), suggesting that *Prph-*eGFP is a useful marker for mature type II SGN perikarya. The unique localization to the distal region of the spiral ganglia (Figure 2) has been reported (Berglund and Ryugo, 1987; Hafidi, 1998; Nayagam et al., 2011; Maison et al., 2016; Grandi et al., 2020); however, we observed that this primarily distal distribution of type II SGNs was not uniform along the cochlea.

Interestingly, while *Prph-*eGFP-positive type II neurons are located primarily in the distal third of the spiral ganglion, there was a broader distribution at the apex than the base or middle turn (Figure 2). This broader distribution in the apex was more similar to the overall distribution of *Prph-*eGFP-positive cells in the vestibular ganglion (Supplementary Figure 1). Like the unmyelinated type II SGNs innervating type II cochlear hair cells, the unmyelinated vestibular afferents also express peripherin (Leonard and Kevetter, 2002). Given that the apex of the cochlea is converted into a vestibular/lagena-like epithelial arrangement with the loss of *N-Myc* (Kopecky et al., 2011), this might suggest an evolutionary relationship between the distribution of *Prph-*eGFP-positive cells in the vestibular ganglion and the spiral ganglion at the apex. However, the significance of the distribution of *Prph-*eGFP-positive perikarya in the two different ganglia is not known. Furthermore, there is heterogeneity in type II SGNs in the specific genes they express depending upon their location in the cochlea (Vyas et al., 2019). Thus, the different genetic makeup of the SGNs located at the base versus the apex may also play a role in their specific distribution, although it remains to be explored.

Remarkably, while *Prph-*eGFP expression became restricted only to type II SGN cell bodies, the expression of *Prph-*eGFP in peripheral processes was not (Figure 3). Similarly, peripherin protein expression in type I SGNs peripheral processes to IHCs has also been shown in adult animals (Hafidi, 1998). Furthermore, the expression is not restricted to only type II peripheral processes as it is to the cell body, but the relative level of *Prph-*eGFP expression in both type I and II SGN peripheral processes changes over time (Figure 3). The expression of *Prph-*eGFP appears to come on as a wave from base to apex after the peripheral processes have innervated the hair cells (Supplementary Figure 2). Before the onset of hearing, *Prph-*eGFP expression decreases in the same base to apex progression, first with the type II SGNs followed by type I SGNs. By P30, minimal *Prph-*eGFP expression in SGN peripheral processes remains (Figure 3). Peripherin protein can be identified in adult rats (Hafidi, 1998); however, the colabeling of peripherin antibodies with other type II SGN markers has shown that some type II SGNs do not express peripherin (Vyas et al., 2017). What remains to be explored is how changes in *Prph-*eGFP expression in type II peripheral processes correlate with potential protein expression changes, especially within different cochlea regions already known to express different genes (Vyas et al., 2019).

Centrally, at E18.5, all three regions of the cochlear nucleus, AVCN, PVCN, and DCN are innervated by *Prph-*eGFP-positive type I and II SGNs (Figures 4, 5). This finding is unsurprising given that even before this stage, lipophilic dye labeling has shown that SGNs reach all areas of the cochlear nucleus (Fritzsch et al., 1997; Schmidt and Fritzsch, 2019; Filova et al., 2020). Interestingly, by P4, central processes to the DCN are no longer positive for *Prph-*eGFP and remain that way through at least 9 months. Whether this has anything to do with the unique function of the DCN from that of the AVCN and PVCN remains to be explored. By 9 months, virtually no *Prph-*eGFP-positive central projections are detected in any of the cochlear nuclei. However, since at 9 months, only the type II SGNs appear to express *Prph-*eGFP in their central processes, and given that these neurons only make up about 5% of the total neuronal population (Spoendlin, 1971), the ability to distinguish their projections may be that they are below the level of reliable fluorescence detection in the brain. More likely, since P30 central processes also appear to be specific to type II SGNs and *Prph-*eGFP expression is detected in the AVCN and PVCN at this stage, the level of *Prph-*eGFP in the central most aspect of the central processes may decrease from P30 to 9 months as it does in peripheral processes over time.

Collectively, these results show that *Prph-*eGFP is a reliable marker for type II SGN cell bodies beyond P8; however, it is not completely specific to peripheral or central processes. Still, type II central processes appear to exclusively express *Prph-*eGFP at P30 and beyond and could serve as a reliable marker in these older animals providing further confirmation of specificity through colabeling with other markers known to be exclusively expressed in mature type II SGNs.

## Data Availability Statement

The raw data supporting the conclusions of this article will be made available by the authors, without undue reservation.

## Ethics Statement

The animal study was reviewed and approved by Institutional Animal Care and Use Committee (IACUC) of The University of Nevada, Reno, and The University of Iowa.

## Author Contributions

KE, JK, and BF designed and performed the experiments and wrote the manuscript. JL, IJ, and GP assisted with data collection. EY and BF conceptualized the manuscript. All authors contributed to the article and approved the submitted version.

## Conflict of Interest

The authors declare that the research was conducted in the absence of any commercial or financial relationships that could be construed as a potential conflict of interest.
