# Supplementary figures and images for: Developmental Changes in Peripherin-eGFP Expression in Spiral Ganglion Neurons
Source: Front Cell Neurosci. 2021 Jun 15;15:678113. doi: 10.3389/fncel.2021.678113 (PMC8239239; doi:10.3389/fncel.2021.678113)

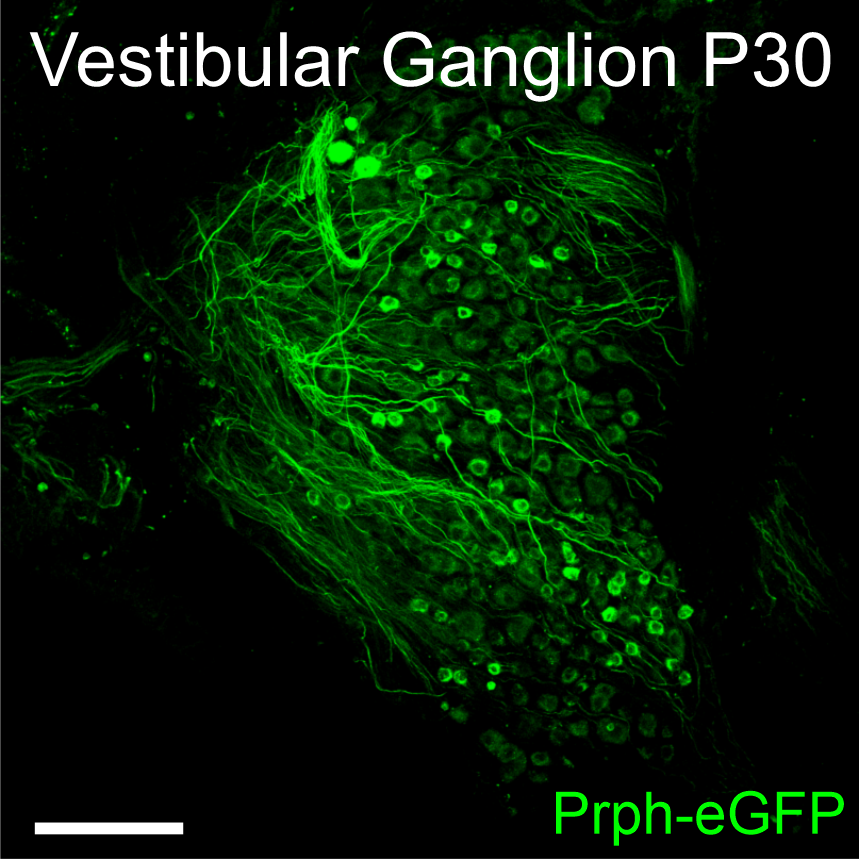

Supplement: Supplementary file 1 [file Image_1.TIF]

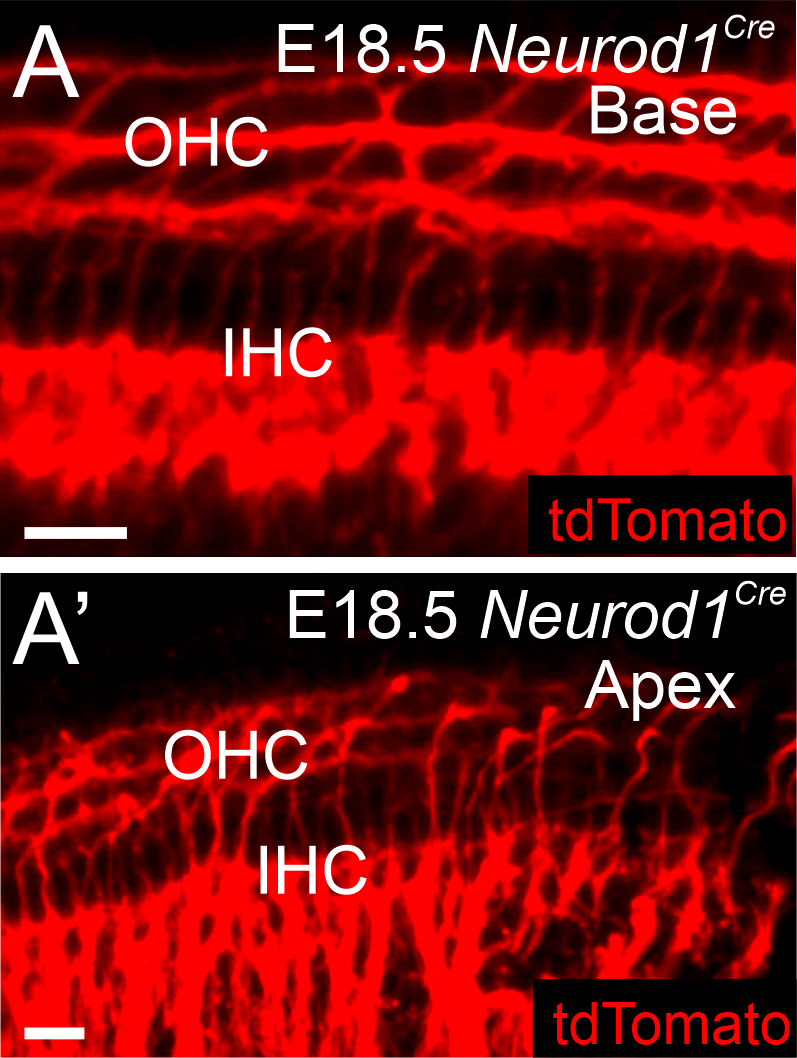

Supplement: Supplementary file 2 [file Image_2.TIF]

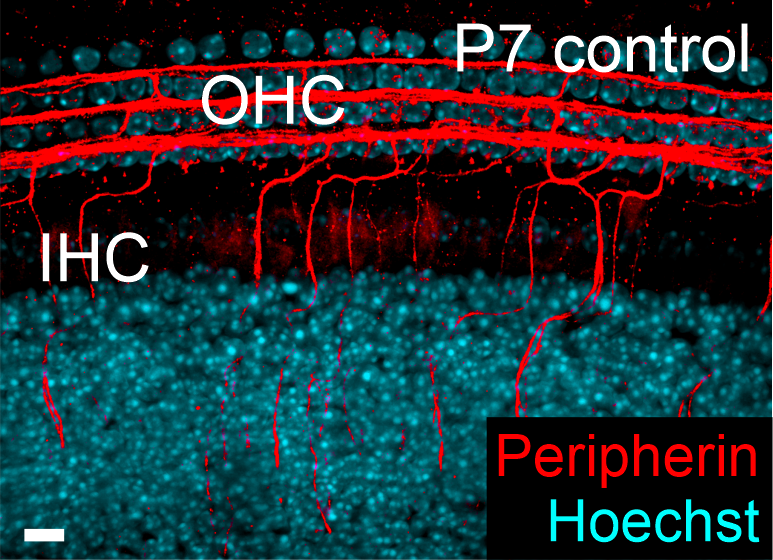

Supplement: Supplementary file 3 [file Image_3.TIF]
