# Supplementary material for: Developmental Changes in Peripherin-eGFP Expression in Spiral Ganglion Neurons
Source: Front Cell Neurosci. 2021 Jun 15;15:678113. doi: 10.3389/fncel.2021.678113 (PMC8239239; doi:10.3389/fncel.2021.678113)
Supplement: Supplementary file 4 [file Data_Sheet_1.DOCX]

**Supplemental Figure 1.** *Prph-*eGFP-positive neurons are distributed throughout the vestibular ganglion at P30. The scale bar is 100 µm.

**Supplemental Figure 2.** Expression of tdTomato specifically in afferents using Neurod1-cre shows both types I and type II peripheral processes are innervating inner hair cells (IHCs) and outer hair cells (OHCs), respectively, in the base (A) and apex (A’) at E18.5. Scale bars are 100µm.

**Supplemental Figure 3.** Peripherin antibody staining at P7 in wild-type mice. The scale bar is 100 µm.
